# Oral and Intestinal Bacterial Flora in Patients with Increased Periodontal Inflamed Surface Area: A Cross-Sectional Study

**DOI:** 10.3390/jcm13133756

**Published:** 2024-06-27

**Authors:** Kentaro Taniguchi, Norio Aoyama, Toshiya Fujii, Sayuri Kida, Tomomi Yata, Aya K. Takeda, Masato Minabe, Motohiro Komaki

**Affiliations:** 1 Department of Periodontology, Kanagawa Dental University, 82 Inaoka-cho, Yokosuka 238-8580, Kanagawa, Japan; tanikentaniken2@gmail.com (K.T.); t.fujii@kdu.ac.jp (T.F.); kdsyr61@gmail.com (S.K.); t.oct.o2m6o@gmail.com (T.Y.); m.komaki@kdu.ac.jp (M.K.); 2 Department of Education Planning, Kanagawa Dental University, 82 Inaoka-cho, Yokosuka 238-8580, Kanagawa, Japan; 3 Cykinso, Inc., 1-36-1 Yoyogi, Shibuya-ku, Tokyo 151-0053, Japan; takeda@cykinso.co.jp; 4 Bunkyou Dori Dental Clinic, 2-4-1 Anagawa, Inage-ku, Chiba 263-0024, Chiba, Japan; minabe-m@wk9.so-net.ne.jp; 5 Department of Environmental Pathology, Kanagawa Dental University, 82 Inaoka-cho, Yokosuka 238-8580, Kanagawa, Japan

**Keywords:** PISA, 16S rRNA amplicon sequencing analysis, microflora

## Abstract

**Background/Objectives**: Periodontitis is caused by bacterial plaque. The oral microflora may interact with the intestinal microflora and play a role in the development of periodontitis. The periodontal inflamed surface area (PISA) has been shown to be a useful indicator of periodontal disease related to systemic diseases; however, few studies have shown an association between PISA and the bacterial flora. This study aimed to determine the association between PISA and oral and intestinal bacteria. **Methods**: Participants were recruited between 2018 and 2021 at the Medical and Dental Collaboration Center of Kanagawa Dental University Hospital. A periodontal clinical examination was performed, and the PISA was calculated. Salivary tests were conducted, and leukocyte scores in the saliva were calculated. Moreover, 16S rRNA amplicon sequencing was performed using saliva and stool samples to analyze oral and intestinal bacteria, respectively. **Results**: Higher PISA levels resulted in an increased presence of *Bacteroides* and a decreased presence of *Proteobacteria* and *Actinobacteria* in the saliva. An increase in *Bacteroides* was detected in the saliva of patients with high leukocyte scores. No correlation was observed between PISA and intestinal bacteria. **Conclusions**: *Bacteroides* was highly abundant in the saliva of patients with worsened periodontal conditions, as indicated by PISA. No association was found between PISA and intestinal bacteria.

## 1. Introduction

Periodontal disease is a chronic inflammatory disease that involves the destruction of the periodontal support tissues around the teeth. Moderate and advanced periodontitis are more common in older individuals, with an incidence of more than 70% in certain populations [1]. Plaque is the main cause of periodontal disease, and various other systemic diseases are associated with periodontal disease [2]. *Porphyromonas gingivalis* (*P. gingivalis*) is a keystone pathogen associated with periodontitis development, a chronic inflammatory pathology characterized by the destruction of the supporting tooth structures. Macrophages are recruited in the inflammatory infiltrate of patients with periodontitis and activated by the bacterial virulence factors, promoting an inflammatory microenvironment characterized by cytokine production, prostaglandins, and metalloproteinases that foster the tissular destruction characteristic of periodontitis [3]. *Fusobacterium nucleatum* was found to cause the production of pro-inflammatory cytokines in the early stages of infection [4]. Oral bacteria survival in the gut shifts the pancreatic, biliary, and liver microbial profiles, which increases the influx of inflammatory mediators into the gut [5]. A large body of evidence suggests that periodontal disease increases the risk of developing and progressing to extra-oral conditions such as diabetes, atherosclerosis, rheumatoid arthritis, and inflammatory bowel disease. In this context, the influx of bacteria and/or bacterial products and inflammatory cytokines derived from inflamed periodontal tissue into the systemic circulation is thought to be the most likely causative mechanism. Moreover, recent studies have shown that oral bacteria, particularly periodontal bacteria, play a role in inducing dysbiosis of the gut microbiota, which in turn induces gut dysbiosis-related pathologies associated with systemic diseases [6]. The oral cavity is one of the largest microbial sites, and the microbiome is stored around the teeth, tongue, soft and hard palates, and gingival sulcus as an important component of oral and tooth-related health and diseases [7]. In recent years, the understanding of oral flora has progressed rapidly. Clinical trials are performed to evaluate the effects of probiotics [8]. Some bacteria are proposed as potential probiotics because some strains can inhibit periodontopathogens and have been reported as safe for humans [9]. Probiotics are a promising option to lower pathogenic bacterial counts in patients undergoing dental therapy [10]. These reports show that the bacteria in the oral cavity seem to be controlled in a certain kind of balance.

Whole-metagenomic sequencing has shown that bacteria, such as *Actinobacteria*, *Proteobacteria*, *Fusobacteria*, *Bacteroides*, and *Firmicutes,* account for approximately 80–95% of the total oral microbiota [11]. In a previous study comparing periodontitis and non-periodontitis groups, the periodontitis group had more *Firmicutes* and *Bacteroides*, whereas the non-periodontitis group had more *Proteobacteria* and *Fusobacteria* [12]. The development of periodontitis is associated with an increase in the number of bacteria belonging to the phylum *Bacteroides*. The phylum *Bacteroides* is one of the most abundant phyla in both the oral cavity and gut [13]. Oral bacteria are classified in the order of their association with periodontal disease, with the bacteria most associated with periodontal disease termed the red-complex [14]. The three bacterial species in the red complex are *P.gingivalis*, *Treponema denticola*, and *Tannerella forsythia*, considered the most significant causes of severe periodontitis; two of these bacteria belong to the phylum *Bacteroides*. Elucidating oral microflora is crucial for understanding both oral and systemic health [15].

The oral cavity, which is the entrance to the gastrointestinal tract, contains a large microbial community that differs from the gut microbiota [16]. Given that large amounts of oral microbiota are constantly swallowed in saliva and food, it is reasonable to assume that oral microbiota may migrate endogenously to the gut, even in the presence of antimicrobial barriers such as gastric acid and bile. The oral and intestinal tracts are interrelated, and both contain rich natural microflora. The gut microbiota interacts with the oral microbiota and may play a role in the development of periodontitis [17].

Periodontal inflamed surface area (PISA) can be determined based on clinical indicators of periodontal disease, such as periodontal pocket depth, clinical attachment level, amount of gingival recession, and bleeding on probing [18,19]. Periodontal ligament tissue rich in blood vessels is present on the periodontal surface, and periodontal disease-associated bacteria can be detected in other organs via the bloodstream from periodontitis lesions [20,21]. Compared to conventional periodontal tissue examination, which indicates the degree of tissue destruction and inflammation, PISA is an indicator that can easily reveal the relation between periodontal disease and the whole body, as periodontal disease-associated bacteria are transported to other organs via the bloodstream.

The relation between the clinical state of periodontal disease and the oral and intestinal bacterial flora is important. However, few reports have shown a relation between PISA and the oral or intestinal microflora. This study aimed to determine the relation between PISA and the oral and intestinal bacteria.

## 2. Materials and Methods

### 2.1. Study Participants

The participants were recruited at the Medical and Dental Collaboration Center of Kanagawa Dental University Hospital between 2018 and 2021. Participants had to be at least 20 years old and agree to participate. The following were excluded: those with an antibiotic intake within the past two months, those with serious systemic infection, those undergoing pregnancy, and those with a lactation status. This study was approved by the Ethics Committee of Kanagawa Dental University School of Dentistry (No. 801) and conducted in compliance with the Declaration of Helsinki. The purpose and procedures of the study were explained to the participants. Written informed consent was obtained from all participants prior to their participation.

### 2.2. Clinical Examinations

General information about the participants, including age and sex, was obtained from the initial interviews and medical records. 

Clinical periodontal status was assessed by a trained periodontist. The number of remaining teeth, excluding wisdom teeth, was counted, and the pocket depth and bleeding on probing were measured using a manual probe (PCP-UNC 15, Hu-Friedy, Chicago, IL, USA) at six points per tooth. These periodontal parameters were used to calculate the PISA using a previously reported method [18].

The leukocyte score in saliva was calculated using Sill-Ha (Arkray, Inc., Kyoto, Japan) according to the manufacturer’s instructions, as previously described [22]. Briefly describing the method of use, the participant held and rinsed the solution in their mouth for 10 s and collected the mixture by spitting it into a cup. The collected saliva was applied to a test strip, which was then placed in a dedicated testing device. The leukocyte count in the saliva was calculated from the saliva test results, which indicated the degree of gingival inflammation. The values range from 0 to 100, with higher values indicating a higher number of leukocytes.

### 2.3. Detection of Bacterial Flora

The oral and intestinal bacteria were examined as previously reported [23,24]. Briefly, the participants rinsed their mouths with 10 mL of sterile saline for 10 s, and saliva samples were collected by spitting into a cup. The collected saliva and fecal samples were transported frozen and at room temperature, respectively, and stored at 4 °C. 16S rRNA amplicon sequencing was performed using saliva samples from oral bacteria and stool samples from intestinal bacteria. Briefly, amplicons of the V1V2 region were prepared using the forward primer 16S_27Fmod (TCG TCG GCA GCG TCA GAT GTG TAT AAG AGA CAG AGR GTT TGA TYM TGG CTC AG) and the reverse primer 16S_338R (GTC TCG TGG GCT CGG AGA TGT GTA TAA GAG ACA GTG CTG CCT CCC GTA GGA GT). The libraries were sequenced in a 250 bp paired-end run using a MiSeq Reagent Kit v2 (Illumina, San Diego, USA; 500 cycles) [25].

### 2.4. Statistical Analysis

The Shapiro–Wilk test was used to test the normality of the data distribution. Numerical data are presented as medians and interquartile ranges. Spearman’s rank correlation coefficient was used to examine correlations between continuous variables. In the correlation analysis, the Bonferroni correction was used. The Wilcoxon signed-rank sum test was used to compare categorical values. The Shapiro–Wilk test showed that the *p*-values for all indicators were less than 0.05. The Wilcoxon signed-rank sum test was used because data did not follow normality in the Shapiro–Wilk test. JMP version 14.2.0 software (SAS, Cary, NC, USA) was used. A *p*-value < 0.05 was considered statistically significant.

## 3. Results

The characteristics of the study participants are listed in Table 1. There were 112 women and 67 men participants, with a total of 179 participants. The median age of the participants was 68 years.

**Figure 1:**
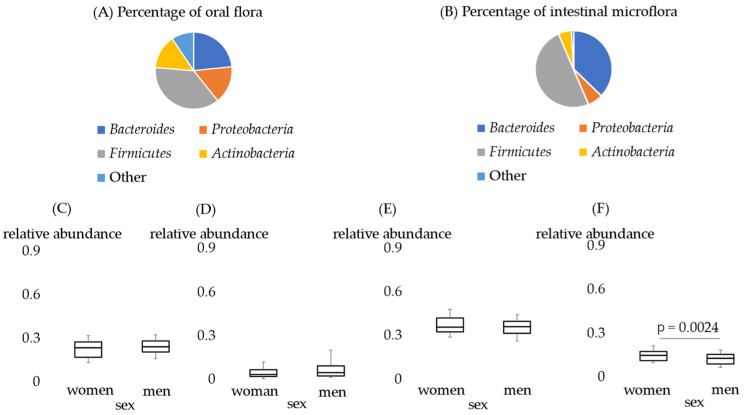
Proportion of bacteria and differences in the proportion of bacteria between men and women. Percentage of bacteria present in oral (**A**) and intestinal samples (**B**), and the proportions of *Bacteroides* (**C**), *Proteobacteria* (**D**), *Firmicutes* (**E**), and *Actinobacteria* (**F**) are shown.

Figure 1A,B shows the proportion of bacteria in the oral and intestinal tracts of the participants. The proportion of *Firmicutes* was high among both oral and intestinal bacteria.

Figure 1C–F shows the proportions of bacteria detected according to sex. The detection rate of *Actinobacteria* was significantly higher in women than in men.

**Figure 2:**
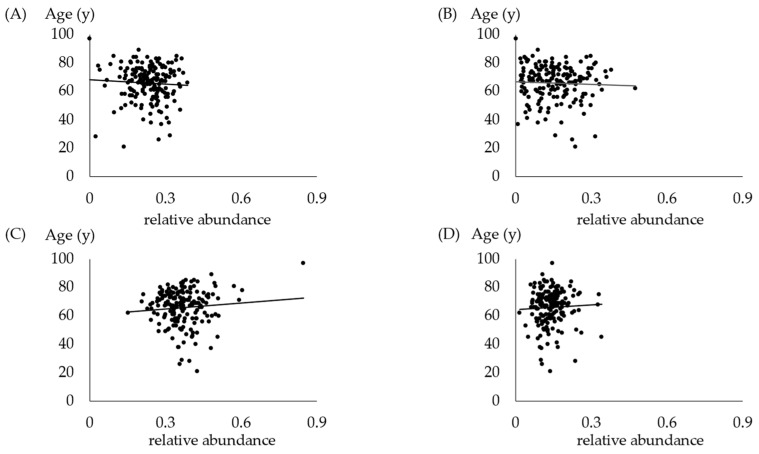
Relation between age and each phylum in oral samples. (**A**) Relation between age and *Bacteroides*. *p* = 0.3357. (**B**) Relation between age and *Proteobacteria*. *p* = 0.6575. (**C**) Relation between age and *Firmicutes*. *p* = 0.7419. (**D**) Relation between age and *Actinobacteria*. *p* = 0.1755.

The association between age and oral bacteria is shown in Figure 2. No correlation was found between age and the presence of oral bacteria.

**Figure 3:**
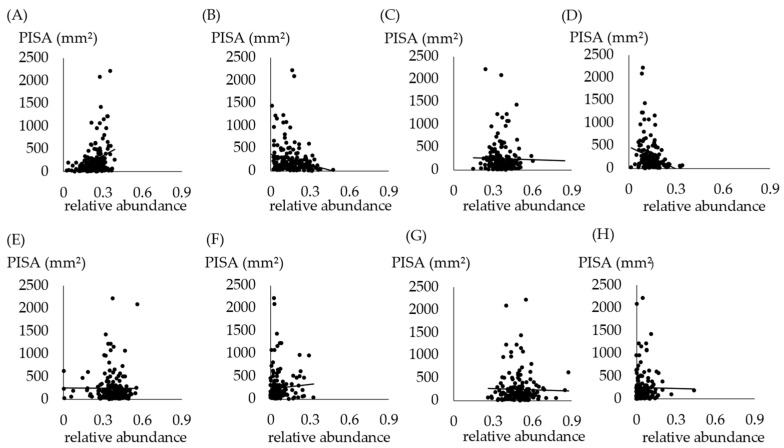
Relation between PISA and each phylum in oral samples and intestinal samples. (**A**) Relation between PISA and *Bacteroides* in oral samples. *p* < 0.0001, ρ = 0.3223. (**B**) Relation between PISA and *Proteobacteria* in oral samples. *p* = 0.0054, ρ = −0.2093. (**C**) Relation between PISA and *Firmicutes* in oral samples. *p* = 0.5667. (**D**) Relation between PISA and *Actinobacteria* in oral samples. *p* = 0.0027, ρ = −0.2254. (**E**) Relation between PISA and *Bacteroides* in intestinal samples. *p* = 0.1971. (**F**) Relation between PISA and *Proteobacteria* in intestinal samples. *p* = 0.4018. (**G**) Relation between PISA and *Firmicutes* in intestinal samples. *p* = 0.8628. (**H**) Relation between PISA and *Actinobacteria* in intestinal samples. *p* = 0.1971.

The association between PISA and oral bacteria is shown in Figure 3A–D. Correlations were observed in *Bacteroides* (A), *Proteobacteria* (B), and *Actinobacteria* (D). A higher PISA resulted in more *Bacteroides* and fewer *Proteobacteria* and *Actinobacteria*. The relation between PISA and intestinal bacteria is shown in Figure 3E–H. No association was observed between PISA and intestinal bacteria.

**Figure 4:**
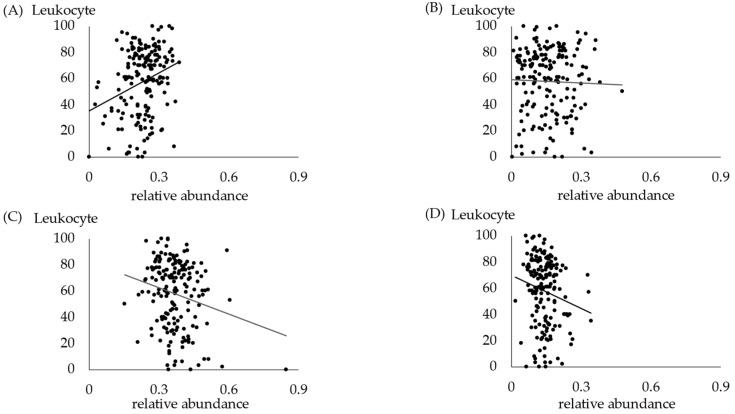
Relation between leukocyte score of salivary tests and each phylum in oral samples. (**A**) Relation between leukocytes and *Bacteroides*. *p* = 0.0025, ρ = 0.2266. (**B**) Relation between leukocytes and *Proteobacteria*. *p* = 0.7436. (**C**) Relation between leukocytes and *Firmicutes*. *p* = 0.0318, ρ = −0.1619. (**D**) Relation between leukocytes and *Actinobacteria*. *p* = 0.0255, ρ = −0.1684.

The association between leukocyte scores in the saliva and bacteria in the oral cavity is shown in Figure 4. Correlations were observed among *Bacteroides*, *Firmicutes*, and *Actinobacteria*. While a negative correlation was found between salivary leukocytes and *Firmicutes* and *Actinobacteria*, this correlation was not significant after Bonferroni correction. Increased *Bacteroides* were found in patients with high leukocyte scores.

**Figure 5:**
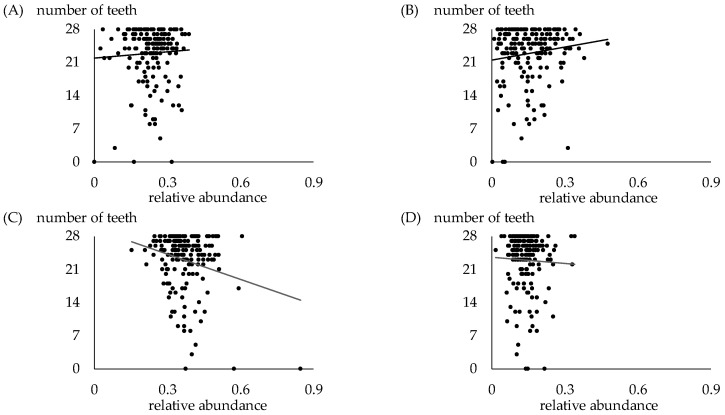
Relation between number of teeth and each phylum in oral samples. (**A**) Relation between number of teeth and *Bacteroides*. *p* = 0.8931. (**B**) Relation between number of teeth and *Proteobacteria*. *p* = 0.1776. (**C**) Relation between number of teeth and *Firmicutes*. *p* = 0.2029. (**D**) Relation between number of teeth and *Actinobacteria*. *p* = 0.3773.

Figure 5 shows the relation between the number of teeth and oral bacteria in the participants. No correlation was found between oral bacteria and the number of teeth.

**Figure 6:**
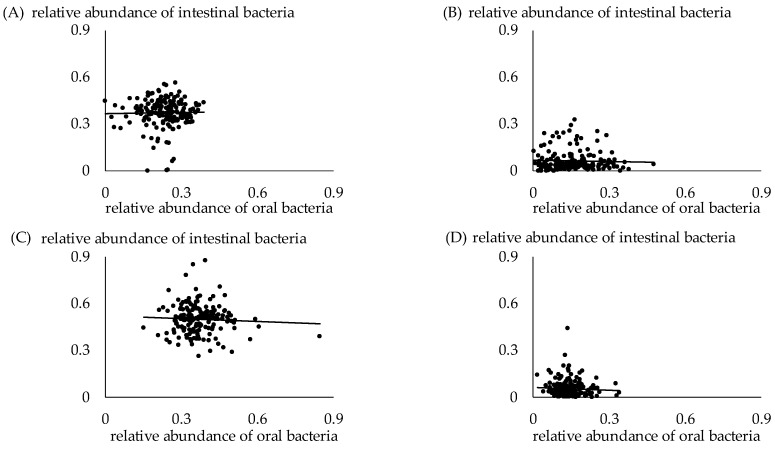
Relation between proportion of same phylum in oral and intestinal samples. (**A**) *Bacteroides*. *p* = 0.7348. (**B**) *Proteobacteria*. *p* = 0.9514. (**C**) *Firmicutes*. *p* = 0.8664. (**D**) *Actinobacteria*. *p* = 0.8720.

The relation between the proportions of the same phylum in the oral and intestinal samples is shown in Figure 6. No correlation was observed between oral and intestinal bacteria belonging to the same phylum.

## 4. Discussion

In this study, an association between PISA and a specific bacterial phylum in saliva was found. The present study showed a positive correlation between PISA and *Bacteroides.* No association was found between PISA and gut bacteria.

PISA is one of the clinical periodontal parameters and is known to be associated with systemic disease [26]. Periodontal disease can be quantified by combining multiple indicators, such as bleeding on probing, tissue color, pocket depth, bone loss, and clinical attachment loss. PISA is an indicator of periodontal disease, and its advantage is its ability to quantify the degree of inflammation in the periodontal tissue with a single value [27]. There have been studies on the association of oral bacteria with clinical parameters, such as bleeding on probing, pocket depth, and clinical attachment loss [28]. Few studies have shown the relation between PISA and oral bacteria; therefore, the association between PISA and oral bacteria was investigated in this study. 

Recent evidence suggests that many uncharacterized host immune factors may be involved in the complex interactions between the oral and extraoral microbiomes [29]. Inflammatory periodontal lesions are primarily caused by heterogeneous bacterial communities, as supported by studies using mouse models of ligature-induced periodontitis [30]. It has been suggested that periodontitis may evolve as a result of ecological perturbations rather than the presence of specific bacterial species [31]. Periodontitis exhibits a polymicrobial pathogenesis within the framework of a complex microbial ecosystem. Advances in sequencing technology have enabled comprehensive studies to elucidate differences in bacterial communities [32]. Moreover, 16S rRNA gene sequencing has characterized the subgingival microbiome and identified health-associated bacteria and bacteria associated with periodontitis [33].

Our results showed a positive correlation between PISA and the phylum *Bacteroides* (Figure 3). Negative correlations with PISA were observed for *Proteobacteria* and *Actinobacteria*. No correlation with PISA was observed for the phylum *Firmicutes*. These results partially agree with those of previous reports [34,35]. The proportion of *Proteobacteria* decreases with increasing depth of the periodontal pockets [34]. It is also shown that periodontitis communities are characterized by an increased relative abundance of *Firmicutes*, while healthy periodontal tissue is characterized by an increased relative abundance of *Actinobacteria* [35].

In this study, no association was found between PISA and intestinal bacteria (Figure 3). Previous studies have shown that in humans, many diseases are associated with changes in the gut microbiota, with increased or decreased abundance of certain bacterial groups [36]. Also, in some animal models, oral administration of *P. gingivalis* to mice leads to significant changes in the gut microbiota, increased serum endotoxaemia, and reduced gut barrier function [37]. Patients with chronic periodontitis have higher levels of *Firmicutes* and *Proteobacteria* and lower levels of *Bacteroides* in the gut microbiota compared to controls [38]. Infection with *Aggregatibacter actinomycetemcomitans*, one of the periodontal pathogens, alters the gut microbiota [39]. Although some papers have shown this relationship between clinical indicators of periodontal disease and gut bacteria, no such association was found in the present study. This may be mainly due to the limited sample size and possible selection bias, as few subjects had severe periodontal disease and most of them were in the early stages of periodontitis. PISA value of more than 130 mm^2^ indicated the presence of periodontitis with 98% sensitivity and 100% specificity; the mean PISA value was highest in the severe periodontitis group (2309 ± 588 mm^2^) and lowest in the healthy group (34 ± 16 mm^2^) [40]. In the future, it is necessary to classify subjects according to the severity of periodontal disease and measure PISA to investigate the association with intestinal bacteria.

The leukocyte score in the saliva test was also associated with specific bacterial phyla (Figure 4). A positive correlation was found between salivary leukocytes and the oral *Bacteroides phylum*. While a negative correlation was found between salivary leukocytes and *Firmicutes* and *Actinobacteria*, this correlation was not significant after Bonferroni correction. Salivary testing can be used as a simple, noninvasive technique to monitor periodontal status and disease progression [41]. According to previous reports, the number and type of oral leukocytes are associated with the severity and extent of periodontal disease [42,43]. Oral polymorphonuclear leukocyte levels have been reported to be independent of leukocyte levels in the peripheral blood and correlated with the degree of inflammation30. Furthermore, significant differences in oral neutrophil counts have been reported between periodontal groups and healthy controls [43]. The estimation of changes in oral salivary leukocyte levels may be useful for monitoring treatment outcomes.

No association was found between oral and intestinal bacteria (Figure 6). According to previous studies, the profiles of the oral and gut microbiota are well separated for the oral–gut barrier [44]. Chronic oral inflammation is also associated with reduced alpha diversity of the gut microbiota, and although several oral taxa were detected in the stool of each patient, there was no clear trend regarding the enrichment of oral taxa [45]. Patients with periodontitis were found to have significantly richer gut flora than those without, but the identification of human oral pathogens was limited [46]. Therefore, oral bacteria may have a small effect on gut bacteria. The results of the present study showed that the oral microbiota was not associated with the gut microbiota. The present study analyzed only at the level of the bacterial phylum and assessed without possible confounding factors such as systemic diseases, medications, dietary intake, and nutrients. Thus, analyses with more detailed subclassifications would be necessary.

This study has several limitations. First, the period of periodontal treatment was not limited; the stage of dental treatment varied among individuals. Second, the study did not include many patients with severe periodontitis because the median PISA score was low. Third, it is difficult to analyze bacteria under the same conditions because they change depending on the living environment and systemic conditions of the participants. This study did not consider the participants’ systemic diseases, medication status, or living environment. According to previous reports, the composition of the oral cavity is dominated by *Proteobacteria*, followed by *Bacteroides*, *Firmicutes*, and *Actinobacteria* [47], which differs from the composition of bacteria in this study as shown in Figure 1A. The composition of the intestine was most frequently *Firmicutes*, followed by *Bacteroides* and *Actinobacteria* [48], which is almost the same as our results shown in Figure 1B. The participants’ systemic diseases, medication status, and living environment may have influenced the results. These issues should be addressed in future studies. The observation of a correlation between PISA and oral flora, even in such participants, is important. In future studies, a detailed analysis of patient conditions and bacterial flora is required.

## 5. Conclusions

PISA was associated with the phyla *Bacteroides*, *Proteobacteria*, and *Actinobacteria* in the oral samples. Patients with high *Bacteroides* counts showed higher PISA and leukocyte scores. Although no association was found between oral and intestinal bacteria in the present study, future studies should be conducted taking into account the influence of possible confounding factors.

## Figures and Tables

**Table 1:** Characteristics of the participants.

Variables	
*n*	179
Sex [woman%]	62.9
Age [years]	68 (58.8, 75.0) ^1^
Number of teeth	25 (21.8, 27.0) ^1^
PISA [mm^2^]	153.5 (67.7, 282.9) ^1^
Leukocyte score in saliva	61.5 (38.3, 77.8) ^1^
Proportion of *Bacteroides* in the oral cavity	24.2 (18.8, 28.1) ^1^
Proportion of *Actinobacteria* in the oral cavity	14.0 (10.6, 16.7) ^1^
Proportion of *Proteobacteria* in the oral cavity	15.0 (9.0, 21.9) ^1^
Proportion of *Firmicutes* in the oral cavity	36.3 (32.2, 41.4) ^1^
Proportion of *Bacteroides* in the intestine	37.8 (33.7, 42.8) ^1^
Proportion of *Actinobacteria* in the intestine	3.8 (1.9, 7.6) ^1^
Proportion of *Proteobacteria* in the intestine	4.3 (2.5, 7.5) ^1^
Proportion of *Firmicutes* in the intestine	50.4 (44.1, 55.3) ^1^

^1^ Data are shown as medians (interquartile ranges). PISA, periodontal inflamed surface area.

## Data Availability

The data presented in this study are available upon request from the corresponding author.
